# Late-follicular androgen and estrogen rise associated with clinical pregnancy in IVF/ICSI with granulosa cell studies

**DOI:** 10.1530/RAF-26-0062

**Published:** 2026-07-30

**Authors:** Rungnapa Sririwichitchai, Chantacha Sitticharoon, Issarawan Keadkraichaiwat, Pailin Maikaew, Suchawadee Chanheng, Somsin Petyim

**Affiliations:** ^1^ Department of Physiology, Faculty of Medicine Siriraj Hospital, Mahidol University, Bangkok, Thailand; ^2^ Department of Obstetrics and Gynecology, Faculty of Medicine Siriraj Hospital, Mahidol University, Bangkok, Thailand

**Keywords:** androstenedione, estradiol, aromatase, steroidogenesis, IVF/ICSI

## Abstract

**Graphical Abstract:**

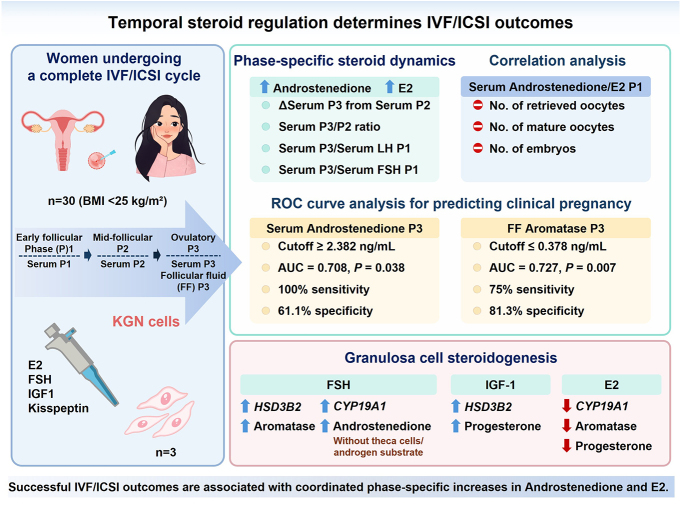

**Abstract:**

This study aimed to characterize phase-specific dynamics of androstenedione and estradiol across IVF/ICSI treatment, identify predictors of reproductive outcomes, and examine the effects of follicle-stimulating hormone (FSH), IGF1, and estradiol on steroidogenesis in human granulosa-like tumor (KGN) cells. In women undergoing IVF/ICSI (10 successful and 19 unsuccessful), serum hormones were measured before recombinant FSH stimulation (Phase 1) and during the mid-follicular (Phase 2) and ovulatory (Phase 3) phases, with follicular fluid (FF) collected at Phase 3. Mechanistic experiments were conducted in KGN cells (*n* = 3) treated for 24 h. Successful pregnancy was associated with greater increases in androstenedione and estradiol from Phase 2 to Phase 3 and higher Phase 3 steroid-to-basal gonadotropin ratios. Elevated Phase 1 androstenedione and estradiol were negatively correlated with the number of retrieved oocytes, mature oocytes, and/or embryos. Phase 3 serum androstenedione (cutoff ≥ 2.382 ng/mL; AUC = 0.708, *P* = 0.038) and FF aromatase (cutoff ≤ 0.378 ng/mL; AUC = 0.727, *P* = 0.007) predicted clinical pregnancy. In KGN cells, IGF1 stimulated early steroidogenesis (*HSD3B2* mRNA expression and progesterone levels), FSH enhanced both early and late pathways (*HSD3B2* and *CYP19A1* mRNA expression together with progesterone, androstenedione, and aromatase levels), and supraphysiological estradiol suppressed downstream steroidogenesis (*CYP19A1* mRNA expression and aromatase and progesterone levels). In conclusion, a favorable pattern with low early-follicular and high late-follicular androstenedione and estradiol was associated with IVF/ICSI success, whereas high early androstenedione and estradiol appeared detrimental. In KGN cells, supraphysiological estradiol without androgen substrate suppressed downstream steroidogenesis, consistent with the adverse effects of excessive early estradiol exposure, supporting granulosa cell contribution to local androgen availability.

**Lay summary:**

Fertility treatment does not always lead to pregnancy. Hormones produced by the ovaries play a key role in pregnancy success. However, it is not clear how hormonal changes over time affect pregnancy. To address this gap, we followed up women undergoing fertility treatment and measured key reproductive hormones, including androstenedione (A4) and estradiol (E2), at different stages of the treatment cycle. We also used human ovarian cells to understand how E2 influences hormone production. We found that women who became pregnant showed a clear rise in A4 and E2 just before ovulation, while women who did not become pregnant had higher hormone levels at the beginning of the cycle. Our study in human ovarian cells supported these results, showing that excessive early A4 and E2 exposure could reduce later hormone production. Our findings may improve the prediction of treatment success and support the development of better fertility care strategies.

## Introduction

The coordinated interplay of hormones across the ovarian cycle is crucial for maintaining reproductive health in humans (Athar *et al.* 2024). Disruptions in hormonal regulation can impair follicular development, oocyte quality, and outcomes (Gavrilovic *et al.* 2021). Infertility continues to increase globally, affecting around 1 in 6 adults (approximately 17.5%) (World Health Organization 2023), and assisted reproductive technologies, such as *in vitro* fertilization (IVF), have become increasingly important (Wang & Sauer 2006). IVF success depends not only on reproductive hormones but also on a complex network of endocrine and paracrine signals (Dabbagh Rezaeiyeh *et al.* 2022).

Androstenedione (A4) is a major androgen precursor of estrogen biosynthesis (Burger 2002). In women undergoing IVF, baseline serum A4 has been proposed as a predictor of ovarian response to stimulation (Garzia *et al.* 2022), and a higher day 4 serum A4 level correlates with higher fertilization rates in insulin-resistant women (Sathyapalan *et al.* 2017). In contrast, baseline serum A4 was lower in women who achieved clinical pregnancy (Kahyaoglu *et al.* 2023), and a lower follicular fluid (FF) A4 level at oocyte retrieval has been associated with live birth (Kushnir *et al.* 2016).

Estradiol (E2) is essential for follicular growth, oocyte maturation, and endometrial receptivity (Anifandis *et al.* 2005, Chauvin *et al.* 2022). Evidence from both Asian and Western populations indicates that optimal serum E2 levels on the day of human chorionic gonadotropin (hCG) administration are associated with a greater number of retrieved oocytes, better oocyte quality, improved endometrial receptivity, and better pregnancy outcomes (Blazar *et al.* 2004, Joo *et al.* 2010, Rehman *et al.* 2020). A higher serum E2 level in the late follicular and ovulatory phases has also been associated with improved IVF outcomes. In contrast, excessively high E2 levels at hCG triggering have been linked to reduced implantation and pregnancy rates (Simón *et al.* 1995, Yu Ng *et al.* 2000).

Together, current evidence indicates that A4 and E2 show time- and dose-dependent associations with reproductive outcomes and follicular function. However, integrated phase-specific evaluations linking steroid dynamics with gonadotropin relationships within the same IVF setting remain limited.

This study aimed to compare women with successful and unsuccessful pregnancies by evaluating serum and FF levels of A4, aromatase, and E2 across IVF with intracytoplasmic sperm injection (IVF/ICSI) phases. We analyzed phase-specific differences, Δ changes, fold changes, and steroid-to-gonadotropin ratios and determined predictors of reproductive outcomes. In parallel, experiments in human granulosa-like tumor (KGN) cells investigated the mechanistic effects of E2 under varying hormonal conditions on steroidogenesis. By integrating clinical steroid dynamics with cellular findings, this study aims to inform hormone-based biomarkers and strategies to improve reproductive outcomes.

## Materials and methods

### Ethics approval

The protocol for the IVF/ICSI treatment study was approved by the Siriraj Institutional Review Board of the Faculty of Medicine Siriraj Hospital, Mahidol University, Thailand (Si 241/2017), in compliance with international guidelines for human research protection, such as the Declaration of Helsinki, the Belmont Report, CIOMS Guidelines, and the International Conference on Harmonization in Good Clinical Practice (ICH-GCP). All participants provided written informed consent before the study. The protocol for the KGN cell experiments was granted an ethics exemption (116/2,560 (exemption)) by the same institutional review board, as no human biological samples were involved.

### Participants

Patients undergoing IVF/ICSI at the Faculty of Medicine Siriraj Hospital between April 2018 and May 2019 were enrolled. The inclusion criteria were age 25–40 years, body mass index <25 kg/m^2^ (non-obese by Asian criteria) (Lim *et al.* 2017), regular 28- to 31-day menstrual cycles, and intact bilateral ovaries. The exclusion criteria included poor ovarian response, recent hormonal contraceptive use (<2 months), polycystic ovary syndrome (PCOS), moderate–severe endometriosis, and endocrine disorders (hypo-/hyperthyroidism, Cushing’s syndrome, diabetes, or congenital adrenal hyperplasia). Most couples underwent IVF/ICSI for unexplained infertility. Although detailed sperm parameters and male factor infertility data were not collected as part of the study protocol, severe male factor infertility was not a predominant indication for treatment in this cohort, and no cases required testicular sperm extraction (TESE).

Of 30 participants completing one IVF/ICSI cycle, 10 achieved clinical pregnancy and were classified as the successful pregnancy group, whereas 19 participants did not achieve biochemical pregnancy and were therefore classified as the unsuccessful pregnancy group. Participants with biochemical pregnancy without subsequent clinical pregnancy were not included in either outcome group because hormonal profiles in this subgroup could not be clearly categorized. One participant with ectopic pregnancy was excluded from outcome comparisons but included in correlation analyses according to the statistical plan.

### IVF/ICSI treatment protocol

Participants underwent a complete IVF/ICSI cycle according to a previously published protocol (Qin *et al.* 2021). On cycle day 2–3 (before recombinant follicle-stimulating hormone (rFSH) stimulation; Phase 1), blood samples were collected to assess baseline hormonal levels, and transvaginal ultrasonography (SSD-3500SX, Aloka) was performed to evaluate uterine and ovarian status. Participants then received daily subcutaneous rFSH (GONAL-f, Merck Serono, Germany) for 11 days to induce multifollicular development.

After 6 days of stimulation, a gonadotropin-releasing hormone (GnRH) antagonist (0.25 mg Cetrotide, Merck Serono, Germany) was administered daily to prevent a premature luteinizing hormone (LH) surge. Around day 8 of stimulation (mid-follicular phase; Phase 2), serum hormones were measured and follicular growth was monitored by ultrasound.

On approximately cycle day 12, hCG (Ovidrel, Merck Serono, Germany) was injected to trigger oocyte maturation. Approximately 36 h later (about cycle day 14; ovulatory phase; Phase 3), blood samples were collected before ultrasound-guided oocyte pick-up under intravenous sedation. FF was obtained after retrieval. All IVF cycles in this study were performed using ICSI. All embryo transfers in the present study were performed using day 3 cleavage-stage embryos according to the standard fresh embryo transfer protocol at our institution. Blastocyst-stage (day 5) transfer was mainly performed in frozen embryo transfer cycles, particularly in patients undergoing preimplantation genetic testing for aneuploidy (PGT-A). However, such patients were not included in the present study cohort. Two embryos were transferred when at least two viable embryos were available; otherwise, a single embryo was transferred. Among the 30 participants, 28 received transfer of two embryos, whereas 2 received transfer of a single embryo, including 1 participant in the successful pregnancy group and 1 participant in the unsuccessful pregnancy group. Embryo transfer procedures were performed by a single experienced operator to minimize procedural variability. Serum hCG was measured about 15 days after transfer to confirm biochemical pregnancy, and clinical pregnancy was defined by detection of a fetal heartbeat around cycle day 42.

### KGN cell experiments

KGN cells (RIKEN Bioresource Center, Japan) were cultured in a 1:1 mixture of Dulbecco’s modified Eagle’s medium and Ham’s F-12 nutrient mixture (DMEM/F-12; Gibco, USA), supplemented with 10% fetal bovine serum (FBS) and 1% penicillin–streptomycin (P/S) (complete medium), at 37°C with 5% CO_2_, as previously described (Qin *et al.* 2021).

For each experiment, three independent biological experiments (*n* = 3) were performed. A total of 250,000 cells/well were seeded in 96-well plates in complete medium and maintained for 24 h to allow cell attachment. The medium was subsequently replaced with the corresponding treatment media prepared in serum-free DMEM/F-12 containing 1% P/S, including serum-free control medium, solvent control (0.01% ethanol), E2 alone (0.5, 1.0, or 2.0 μg/mL), follicle-stimulating hormone (FSH) alone (0.01 nM), insulin like growth factor 1 (IGF1) alone (0.01 nM), and combinations of FSH and/or IGF1 with each E2 concentration. Baseline androgen concentrations in the culture medium and FBS supplement were not measured prior to experimentation.

E2 concentrations of 0.5, 1.0, and 2.0 μg/mL were selected based on previous evidence demonstrating inhibitory effects of E2 (0.25, 0.5, 1.0, and 2.0 μg/mL) on basal and hCG-stimulated progesterone (P4) production in human granulosa-luteal cells (Jalkanen 1990). To determine whether KGN cells can synthesize A4 in the absence of theca cells and androgen substrates, a separate experiment was conducted, in which cells were treated with serum-free control medium, kisspeptin alone (10 μM), or FSH alone (0.01 nM) for 24 h.

### RNA extraction and real-time polymerase chain reaction (real-time PCR)

Total ribonucleic acid (RNA) was extracted from KGN cells using TRIzol reagent (Invitrogen, USA) in accordance with the manufacturer’s instructions. Subsequently, 500 ng of RNA were reverse transcribed into complementary deoxyribonucleic acid (cDNA) using the iScript cDNA Synthesis Kit (Bio-Rad, USA) in a thermal cycler under the conditions recommended by the manufacturer.

Quantitative real-time PCR was performed using QPCR Green Master Mix LRox, 2X (Biotechrabbit, Germany) on a CFX96 Real-Time PCR Detection System (Bio-Rad, USA). Hypoxanthine phosphoribosyltransferase 1* (HPRT1)*, which exhibits stable expression in human granulosa cells (Lv *et al.* 2017), was used as the reference gene. Primer sequences for *HPRT1*, cytochrome P450 family 19 subfamily A member 1* (CYP19A1)*, hydroxy-delta-5-steroid dehydrogenase, 3 beta- and steroid delta-isomerase 2* (HSD3B2)*, and hydroxysteroid 17-beta dehydrogenase 1* (HSD17B1)* are listed below:

*HPRT1* (Qin *et al.* 2021):

Forward: 5′ CCT GGC GTC GTG ATT AGT GA 3′.
Reverse: 5′ CCA TCT CCT TCA TCA CAT CTC G 3′.

*CYP19A1* (Qin *et al.* 2021):

Forward: 5′ AGC AAG TCC TCA AGT ATG TTC CAC 3′.
Reverse: 5′ GTC CAC ATA GCC CGA TTC ATT 3′.

*HSD3B2*:

Forward: 5′ TGG TGG AAG AGA AGG AAC TGA 3′.
Reverse: 5′ TAG CTG GGT ACC TTT CAC ATT G 3′.

*HSD17B1*:

Forward: 5′ AGC GTG GGA GGA TTG ATG G 3′.
Reverse: 5′ AAG ACC TCC GCC ACC TCC T 3′.

All primers were designed by the authors, including those previously reported (Qin *et al.* 2021). For each gene, primers were designed to span exon–exon junctions (in either the forward or reverse primer) to ensure specific amplification of cDNA derived from RNA and to minimize genomic DNA amplification. Primer specificity was verified using the NCBI Basic Local Alignment Search Tool (BLAST).

PCR amplification was performed with an initial uracil-DNA glycosylase treatment at 50°C for 2 min, followed by polymerase activation at 95°C for 3 min and 40 amplification cycles of denaturation at 95°C for 15 s and annealing for 30 s at gene-specific temperatures (57°C for *HSD17B1*, 59°C for *HPRT1* and *HSD3B2*, and 62°C for *CYP19A1*). Each reaction was performed in duplicate, and no-template controls were included to detect contamination. Relative gene expression was calculated using the 2^−ΔCT^ method.

### Hormonal assays

In accordance with the institutional recommendations for research specimen processing in faculty-funded research projects, serum anti-Müllerian hormone (AMH), LH, FSH, E2, and P4 concentrations were measured at the central laboratory of the Department of Clinical Pathology, Faculty of Medicine Siriraj Hospital, Mahidol University, using electrochemiluminescence immunoassay (ECLIA; COBAS, Roche Diagnostics GmbH, Germany). According to the manufacturer, assay detection ranges were 0.01–23 ng/mL for AMH, 0.1–200 mIU/mL for LH and FSH, 0.005–3.0 ng/mL for E2, and 0.05–60 ng/mL for P4.

Meanwhile, E2 and P4 measurements in FF samples were not included in the routine institutional laboratory services. In addition, FF specimens were available only in limited volumes, and submission of these samples to the central laboratory would have left insufficient remaining sample volume for additional analyte measurements in the present study. Furthermore, several analytes evaluated in the present study, including A4, IGF1, kisspeptin, and measurements in FF and KGN cell culture supernatants, were not available as routine institutional laboratory services. Therefore, these analytes and specimen types were subsequently analyzed using commercially available enzyme-linked immunosorbent assay (ELISA) or enzyme immunoassay (EIA) kits.

A4 concentrations in serum and FF were measured using ELISA kits (DiaMetra, Italy) with a detection range of 0–10 ng/mL, a minimum detectable concentration of 0.01 ng/mL, and intra-assay coefficients of variation of 3.42% (serum) and 6.89% (FF). Cross-reactivity of the anti-androstenedione antibody was 0.05% for 5α-dihydrotestosterone and DHEA, 0.04% for epitestosterone, 0.027% for DHEA-S, 0.008% for cortisol, 0.007% for P4 and estrone, and <0.001% for testosterone, 17β-estradiol, estriol, and aldosterone.

IGF1 concentrations in serum and FF were also measured using ELISA kits (Elabscience, USA) with a detection range of 1.56–100 ng/mL, a minimum detectable concentration of 0.94 ng/mL, and intra-assay coefficients of variation of 8.70% (serum) and 5.09% (FF), with no significant cross-reactivity or interference from related IGF1 analogs observed. Because the assay procedure did not include a dissociation step from IGF-binding proteins, the measured IGF1 concentrations may have been influenced by endogenous IGF-binding proteins and therefore may not accurately represent total IGF1 levels.

E2 and P4 levels in FF and culture supernatants, and kisspeptin levels in serum and FF, were quantified using EIA kits (Phoenix Pharmaceuticals Inc., USA). Detection ranges were 0–1,000 pg/mL for E2, 0–50 ng/mL for P4, and 0–100 ng/mL for kisspeptin. The minimum detectable concentrations were 10 pg/mL for E2, 0.5 ng/mL for P4, and 0.05 ng/mL for kisspeptin, with an assay sensitivity of 0.08 ng/mL for kisspeptin. Intra-assay coefficients of variation were 8.94% (FF) and 9.81% (supernatant) for E2, 8.0% (FF) and 9.54% (supernatant) for P4, and 6.46% for kisspeptin in serum and FF. Cross-reactivity of the E2 antibody was 2.10% for estrone, 1.50% for estriol, and 0.30% for 17α-estradiol, whereas cross-reactivity with cortisol, cortisone, P4, testosterone, DHEA-sulfate, and 5α-dihydrotestosterone was <0.01%. Cross-reactivity of the P4 antibody was 100% for P4, 0.74% for corticosterone, 0.11% for cortisone, 0.10% for testosterone, 0.086% for androsterone, 0.08% for estrone, 0.075% for prednisolone, <0.08% for cholesterol, <0.024% for estriol, and <0.01% for E2. For kisspeptin, cross-reactivity was 100% for Metastin 1–54 (KiSS-1(68–121))-amide (human), Metastin 45–54-amide (human), RFRP-1 (human), and RFRP-1 (mouse/rat), 4% for NPAF (human), 2% for NPFF (NPSF) (human), FMRF-amide, and KiSS-1 (110–119)-amide (mouse), and 0% for RFRP-3 (human). All assays were performed according to the manufacturers’ instructions, and samples were analyzed in duplicate when feasible.

### Statistical analysis

Statistical analyses were performed using Statistical Package for the Social Sciences (SPSS), version 31. Data normality was assessed with the Kolmogorov–Smirnov test. In the human study, differences between the successful and unsuccessful pregnancy groups across IVF/ICSI phases were analyzed using two-way analysis of variance (ANOVA), and between-group comparisons were performed with independent *t*-tests or corresponding nonparametric tests when appropriate. Changes across treatment phases were evaluated using repeated-measures ANOVA with Fisher’s least significant difference post hoc analysis. Associations between variables were examined using Pearson correlation coefficients. Receiver operating characteristic curve (ROC) analysis was used to assess the predictive performance of A4, aromatase, and E2 at each phase by calculating the area under the curve (AUC), sensitivity, specificity, positive predictive value (PPV), negative predictive value (NPV), and optimal cutoff values (Youden’s index). Odds ratios with 95% confidence intervals were calculated from 2 × 2 contingency tables, and associations were tested using chi-square or Fisher’s exact tests, as appropriate. For KGN cell experiments, treatment effects were analyzed by one-way ANOVA followed by Tukey’s post hoc test. All tests were two-sided, and *P* < 0.05 was considered statistically significant.

## Results

### Human study

#### Comparisons of A4, aromatase, and E2 between the successful and unsuccessful pregnancy groups as well as among different phases of IVF/ICSI treatments

**Figure 1:**
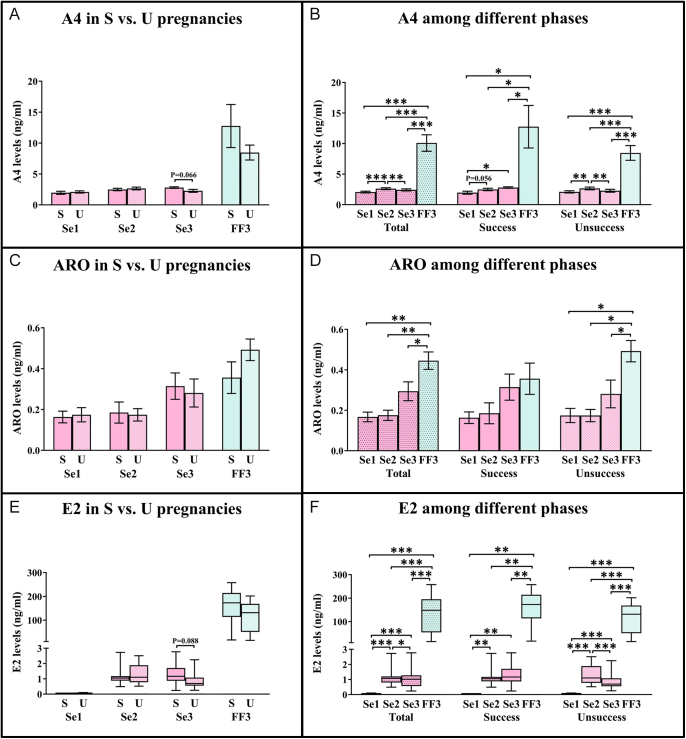
Comparisons between the successful and unsuccessful pregnancy groups as well as across different phases of IVF/ICSI treatment. (A and B) Comparisons of androstenedione (A4), (C and D) aromatase (ARO), and (E and F) estradiol (E2) levels between the successful and unsuccessful pregnancy groups and among different phases of IVF/ICSI treatment. Data are presented as mean ± SEM, and statistical significance between groups is indicated by asterisk (*P < 0.05, **P < 0.01, ***P < 0.001). S, successful pregnancy group; U, unsuccessful pregnancy group; Se1, serum in Phase 1 (the beginning of recombinant FSH (rFSH) stimulation); Se2, serum in Phase 2 (around 8 days after rFSH stimulation); Se3, serum in Phase 3 (on the day of ovum pick-up (OPU)); FF3, follicular fluid in Phase 3.

Data are presented as mean ± SEM for A4 (Fig. 1A and B) and aromatase (Fig. 1C and D) and as median with interquartile range (IQR) for E2 (Fig. 1E and F). Given that serum E2 levels have been previously presented as bar graphs (Qin *et al.* 2021), E2 levels in both serum and FF are presented as box plots in the present study to depict the data distribution (median and IQR) and to avoid redundant graphical representation.

When comparing the successful and unsuccessful pregnancy groups, serum A4 (Fig. 1A; *P* = 0.066) and E2 (Fig. 1E; *P* = 0.088) levels in Phase 3 tended to be higher in the successful pregnancy group.

When compared across different phases, FF A4 levels in Phase 3 were significantly higher than serum A4 levels in Phases 1–3 in total participants (*P* < 0.001 all), the successful pregnancy group (*P* < 0.05 all), and the unsuccessful pregnancy group (*P* < 0.001 all); serum A4 levels in Phase 2 were significantly higher than those in Phases 1 and 3 in total participants (*P* < 0.001 all) and the unsuccessful pregnancy group (*P* < 0.01 all) and tended to be higher than those in Phase 1 in the successful pregnancy group (*P* = 0.056); while serum A4 levels in Phase 3 were significantly higher than those in Phase 1 in successful patients (*P* = 0.043) (Fig. 1B).

Serum aromatase levels were measurable in 4 of 10 samples from the successful pregnancy group and 9 of 19 samples from the unsuccessful pregnancy group, whereas the remaining samples were below the detection range (0.16–10 ng/mL) of the Human ARO (aromatase) ELISA kit. Despite this limitation, FF aromatase levels in Phase 3 were significantly higher than serum levels in Phases 1–3 in total and unsuccessful patients (*P* < 0.05 all) (Fig. 1D).

For E2, FF E2 levels in Phase 3 were significantly higher than serum E2 levels in Phases 1–3 in total participants (*P* < 0.001 all), the successful pregnancy group (*P* < 0.01 all), and the unsuccessful pregnancy group (*P* < 0.001 all) (Fig. 1F). Serum E2 levels in Phase 2 were significantly higher than those in Phases 1 and 3 in total (*P* < 0.05 all) and unsuccessful (*P* < 0.001 all) patients and were significantly higher than those in Phase 1 in successful patients (*P* = 0.005); in addition, serum E2 levels in Phase 3 were significantly higher than those in Phase 1 in total (*P* < 0.001), successful (*P* = 0.005), and unsuccessful (*P* < 0.001) patients (Fig. 1F).

#### Comparisons of phase-specific alterations in A4 and E2 levels during IVF/ICSI treatment between the successful and unsuccessful pregnancy groups

**Figure 2:**
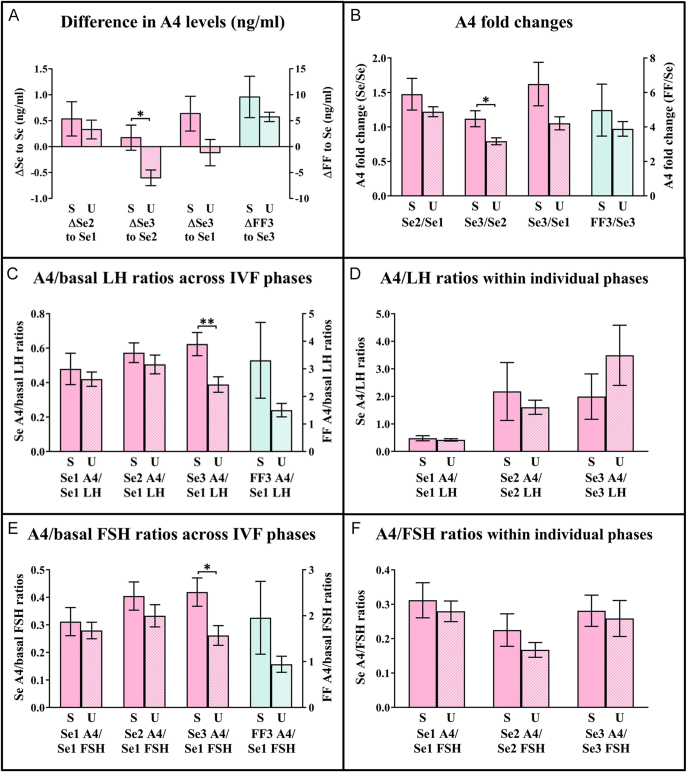
Phase-specific alterations in androstenedione (A4) and A4-to-gonadotropin ratios across IVF/ICSI treatment. (A) Differences in A4 levels (Δserum − serum and ΔFF − serum); (B) fold changes in A4 levels (serum to serum and FF to serum); (C) ratios of A4 to basal LH across IVF/ICSI treatment phases; (D) ratios of A4 to LH within individual phases; (E) ratios of A4 to basal FSH across IVF/ICSI treatment phases; and (F) ratios of A4 to FSH within individual phases. Data are presented as mean ± SEM, and statistical significance between groups is indicated by an asterisk (*P < 0.05). S, successful pregnancy group; U, unsuccessful pregnancy group; Se1, serum in Phase 1 (the beginning of recombinant FSH (rFSH) stimulation); Se2, serum in Phase 2 (around 8 days after rFSH stimulation); Se3, serum in Phase 3 (on the day of ovum pick-up (OPU)); FF3, follicular fluid in Phase 3.

The absolute difference (Fig. 2A, *P* = 0.010) and the fold change in serum A4 levels from Phase 3 to Phase 2 (Fig. 2B, *P* = 0.028) were significantly higher in the successful pregnancy group than in the unsuccessful pregnancy group. In addition, the ratios of serum A4 in Phase 3 to serum LH in Phase 1 (Fig. 2C, *P* = 0.007) and serum A4 in Phase 3 to serum FSH in Phase 1 (Fig. 2E, *P* = 0.019) were significantly higher in the successful pregnancy group than in the unsuccessful pregnancy group.

**Figure 3:**
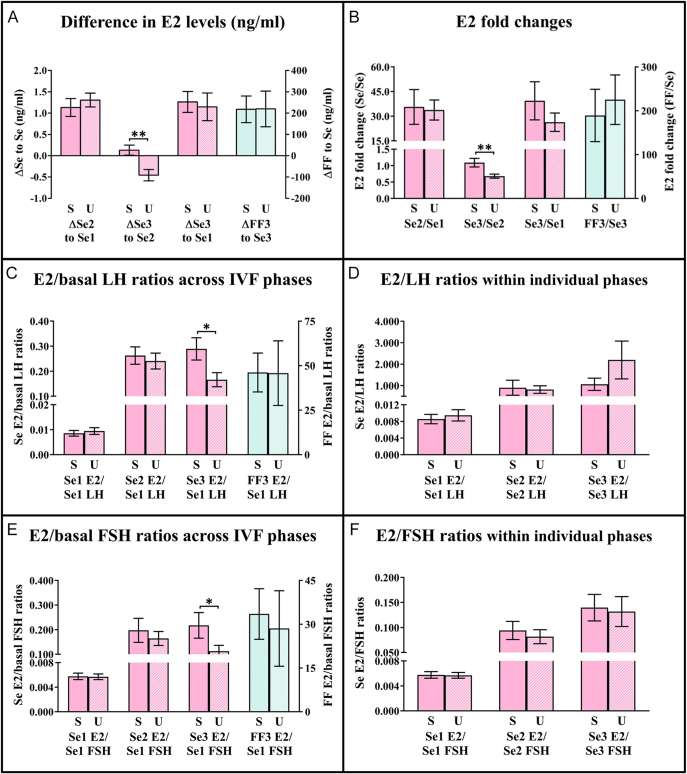
Phase-specific alterations in estradiol (E2) and E2-to-gonadotropin ratios across IVF/ICSI treatment. (A) Differences in E2 levels (Δserum − serum and ΔFF − serum); (B) fold changes in E2 levels (serum to serum and FF to serum); (C) ratios of E2 to basal LH across IVF/ICSI treatment phases; (D) ratios of E2 to LH within individual phases; (E) ratios of E2 to basal FSH across IVF/ICSI treatment phases; and (F) ratios of E2 to FSH within individual phases. Data are presented as mean ± SEM, and statistical significance between groups is indicated by an asterisk (*P < 0.05). S, successful pregnancy group; U, unsuccessful pregnancy group; Se1, serum in Phase 1 (the beginning of recombinant FSH (rFSH) stimulation); Se2, serum in Phase 2 (around 8 days after rFSH stimulation); Se3, serum in Phase 3 (on the day of ovum pick-up (OPU)); FF3, follicular fluid in Phase 3.

Similarly, the absolute difference (Fig. 3A, *P* = 0.008) and the fold change in serum E2 levels from Phase 3 to Phase 2 (Fig. 3B, *P* = 0.003) were significantly higher in the successful pregnancy group than in the unsuccessful pregnancy group. Furthermore, the ratios of serum E2 in Phase 3 to serum LH in Phase 1 (Fig. 3C, *P* = 0.022) and serum E2 in Phase 3 to serum FSH in Phase 1 (Fig. 3E, *P* = 0.043) were significantly higher in the successful pregnancy group than in the unsuccessful pregnancy group.

#### Correlations between two factors in total, successful, and unsuccessful patients undergoing IVF/ICSI treatment

Correlations of A4, aromatase, and E2 with hormonal parameters and outcomes in total, successful, and unsuccessful patients undergoing IVF/ICSI treatment are presented in Tables 1, 2, 3, respectively.

**Table 1:** Correlations between androstenedione (A4) and hormonal parameters and outcomes in total, successful, and unsuccessful patients undergoing IVF/ICSI treatment. Data are presented as correlation coefficients (*r*) and corresponding *P*-values.

Parameter/variable	Total (*n* = 30)		Successful (*n* = 10)		Unsuccessful (*n* = 19)	
	*r*	*P*-value	*r*	*P*-value	*r*	*P*-value
Serum A4 Phase 1						
Serum FSH Phase 1	−0.304	0.109	0.097	0.790	−0.497	0.036*
Serum A4 Phase 2	0.625	<0.001^‡^	0.090	0.804	0.820	<0.001^‡^
Serum A4 Phase 3	0.397	0.044*	0.162	0.702	0.597	0.011*
Serum aromatase Phase 1	−0.430	0.020*	0.090	0.804	−0.595	0.009^†^
Serum aromatase Phase 2	−0.441	0.024*	−0.231	0.550	−0.562	0.023*
Serum P4 Phase 2	0.450	0.018*	0.250	0.516	0.537	0.026*
Serum P4 Phase 3	0.150	0.446	−0.700	0.036*	0.387	0.113
Serum E2 Phase 3	−0.003	0.987	−0.502	0.168	0.509	0.037*
No. of matured oocytes	−0.221	0.299	−0.694	0.038*	0.270	0.279
Serum A4 Phase 2						
Serum AMH Phase 1	0.366	0.060	0.706	0.033*	0.467	0.059
Serum LH Phase 2	0.419	0.026*	0.469	0.203	0.378	0.122
Serum LH Phase 3	0.357	0.080	−0.234	0.577	0.538	0.032*
Serum A4 Phase 3	0.554	0.003^†^	−0.151	0.722	0.757	<0.001^‡^
Serum P4 Phase 2	0.619	<0.001^‡^	0.400	0.286	0.646	0.004^†^
Serum E2 Phase 2	0.553	0.002^†^	0.709	0.032*	0.556	0.016*
Serum E2 Phase 3	0.457	0.017*	0.450	0.224	0.637	0.006^†^
No. of retrieved oocytes	0.297	0.117	0.647	0.043*	0.379	0.120
No. of matured oocytes	0.536	0.007^†^	0.783	0.013*	0.539	0.038*
Serum A4 Phase 3						
Serum LH Phase 3	0.342	0.095	−0.593	0.122	0.562	0.023*
FF A4 Phase 3	0.603	0.002^†^	0.200	0.704	0.759	0.001^†^
Serum P4 Phase 1	−0.555	0.005^†^	−0.709	0.074	−0.434	0.093
Serum P4 Phase 2	0.326	0.111	−0.050	0.916	0.500	0.041*
Serum E2 Phase 2	0.552	0.003^†^	−0.010	0.981	0.706	0.002^†^
Serum E2 Phase 3	0.613	<0.001^‡^	−0.102	0.809	0.781	<0.001^‡^
FF E2 Phase 3	0.389	0.060	0.886	0.008^†^	0.210	0.435
FF A4 Phase 3						
Serum LH Phase 3	0.420	0.058	−0.045	0.933	0.566	0.035*
Serum E2 Phase 3	0.373	0.073	0.071	0.879	0.585	0.017*
Serum P4 Phase 1	−0.425	0.049*	−0.727	0.064	−0.508	0.063
Serum P4 Phase 2	−0.131	0.562	−0.943	0.005^†^	0.141	0.616

Phase 1, beginning of recombinant FSH (rFSH) stimulation; Phase 2, approximately 8 days after rFSH stimulation; Phase 3, on the day of ovum pick-up (OPU); FF, follicular fluid; AMH, anti-Müllerian hormone; LH, luteinizing hormone; FSH, follicle-stimulating hormone; P4, progesterone; E2, estradiol.

* *P* < 0.05.

^†^ *P* < 0.01.

^‡^ *P* < 0.001.

**Table 2:** Correlations between aromatase and hormonal parameters and outcomes in total, successful, and unsuccessful patients undergoing IVF/ICSI treatment. Data are presented as correlation coefficients (*r*) and corresponding *P*-values.

Parameter/variable	Total (*n* = 30)		Successful (*n* = 10)		Unsuccessful (*n* = 19)	
	*r*	*P*-value	*r*	*P*-value	*r*	*P*-value
Serum aromatase Phase 1						
Serum FSH Phase 1	0.414	0.023*	−0.619	0.056	0.573	0.010*
Serum aromatase Phase 2	0.869	<0.001^‡^	0.800	0.010*	0.893	<0.001^‡^
Serum aromatase Phase 3	0.701	0.005^†^	0.156	0.844	0.847	0.004^†^
Serum P4 Phase 3	−0.417	0.025*	−0.450	0.224	−0.379	0.109
Serum E2 Phase 1	0.394	0.038*	0.043	0.907	0.525	0.030*
Serum E2 Phase 2	0.235	0.229	0.750	0.020*	−0.052	0.839
Serum aromatase Phase 2						
Serum FSH Phase 1	0.089	0.658	−0.767	0.016*	0.524	0.031*
Serum aromatase Phase 3	0.772	0.005^†^	0.944	0.215	0.920	0.003^†^
FF aromatase Phase 3	0.435	0.038*	0.536	0.215	0.257	0.356
Serum E2 Phase 1	0.274	0.186	−0.284	0.459	0.721	0.002^†^
Serum E2 Phase 2	0.180	0.378	0.881	0.004^†^	−0.155	0.554
Serum E2 Phase 3	0.215	0.303	0.857	0.007^†^	−0.093	0.733
No. of retrieved oocytes	0.047	0.815	0.767	0.016*	−0.343	0.177
FF aromatase Phase 3						
Serum FSH Phase 2	0.551	0.004^†^	0.510	0.196	0.500	0.049*
Serum FSH Phase 3	0.582	0.002^†^	0.121	0.774	0.844	<0.001^‡^
No. of embryos	−0.355	0.124	0.033	0.937	−0.808	0.003^†^

Phase 1, beginning of recombinant FSH (rFSH) stimulation; Phase 2, approximately 8 days after rFSH stimulation; Phase 3, on the day of ovum pick-up (OPU); FF, follicular fluid; FSH, follicle-stimulating hormone; P4, progesterone; E2, estradiol.

* *P* < 0.05.

^†^ *P* < 0.01.

^‡^ *P* < 0.001.

**Table 3:** Correlations between estradiol (E2) and hormonal parameters and outcomes in total, successful, and unsuccessful patients undergoing IVF/ICSI treatment. Data are presented as correlation coefficients (*r*) and corresponding *P*-values.

Parameter/variable	Total (*n* = 30)		Successful (*n* = 10)		Unsuccessful (*n* = 19)	
	*r*	*P*-value	*r*	*P*-value	*r*	*P*-value
Serum E2 Phase 1						
Serum AMH Phase 1	−0.530	0.005^†^	−0.276	0.472	−0.649	0.007^†^
Serum LH Phase 2	0.147	0.472	0.512	0.043*	0.120	0.646
Serum LH Phase 3	−0.437	0.033*	−0.786	0.021*	0.512	0.043*
Serum FSH Phase 1	0.643	<0.001^‡^	0.365	0.300	0.615	0.009^†^
Serum FSH Phase 2	0.532	0.004^†^	−0.244	0.496	0.623	0.010*
Serum FSH Phase 3	0.494	0.010*	0.035	0.929	0.599	0.014*
FF P4 Phase 3	0.443	0.030*	0.731	0.040*	0.350	0.201
FF E2 Phase 3	0.333	0.120	0.539	0.168	0.613	0.020*
Serum kisspeptin Phase 1	0.388	0.041*	0.774	0.009^†^	0.123	0.639
No. of retrieved oocytes	−0.484	0.009^†^	−0.440	0.203	−0.619	0.008^†^
No. of matured oocytes	−0.409	0.047*	−0.378	0.315	−0.474	0.075
No. of embryos	−0.634	0.001^†^	−0.631	0.051	−0.740	0.006^†^
Serum E2 Phase 2						
Serum A4 Phase 1	−0.313	0.137	−0.130	0.760	−0.558	0.031*
Serum kisspeptin Phase 1	−0.473	0.011*	−0.700	0.036*	−0.386	0.114
Serum P4 Phase 1	−0.555	0.005*	−0.709	0.074	−0.434	0.093
Serum P4 Phase 2	0.326	0.111	−0.050	0.916	0.500	0.041*
Serum E2 Phase 3	0.613	<0.001^‡^	−0.102	0.809	0.781	<0.001^‡^
FF E2 Phase 3	0.389	0.060	0.886	0.008^†^	0.210	0.435
Serum E2 Phase 3						
Serum P4 Phase 1	−0.378	0.068	−0.253	0.545	−0.538	0.039*
Serum kisspeptin Phase 1	−0.424	0.025*	−0.628	0.070	−0.168	0.505
FF E2 Phase 3						
Serum AMH Phase 1	−0.181	0.408	−0.107	0.819	−0.617	0.014*
Serum FSH Phase 1	0.118	0.573	−0.286	0.493	0.544	0.029*
Serum FSH Phase 3	0.297	0.149	−0.310	0.456	0.527	0.036*
Serum P4 Phase 1	−0.445	0.038*	−0.873	0.010*	−0.324	0.240
Serum P4 Phase 2	−0.461	0.027*	−0.500	0.253	−0.418	0.107
FF kisspeptin Phase 3	−0.361	0.083	0.508	0.245	−0.628	0.009^†^

Phase 1, beginning of recombinant FSH (rFSH) stimulation; Phase 2, approximately 8 days after rFSH stimulation; Phase 3, on the day of ovum pick-up (OPU); FF, follicular fluid; AMH, anti-Müllerian hormone; LH, luteinizing hormone; FSH, follicle-stimulating hormone; P4, progesterone.

* *P* < 0.05.

^†^ *P* < 0.01.

^‡^ *P* < 0.001.

#### ROC curve analysis of A4, aromatase, and E2 for clinical pregnancy prediction

Serum A4 in Phase 3, at a cutoff of ≥ 2.382 ng/mL, demonstrated significant discriminatory performance (AUC = 0.708, *P* = 0.038) (Table 4).

**Table 4:** Receiver operating characteristic (ROC) curve analysis of biomarkers for clinical pregnancy prediction.

Biomarker	Cutoff (ng/mL)	AUC		*P*-value	SN (%)	SP (%)	PPV		NPV		Odds ratio		FET (two-sided)
		Value	95% CI				Value	95% CI	Value	95% CI	Value	95% CI	
Serum A4 Phase 1	≤2.548	0.544	0.318–0.771	0.700	90.0	33.3	42.9	24.5–63.5	85.7	48.7–97.4	4.5	0.46–44.29	0.364
Serum A4 Phase 2	≤3.425	0.561	0.348–0.774	0.573	100.0	33.3	45.5	26.9–65.3	100.0	61.0–100.0	–	–	–
Serum A4 Phase 3	≥2.382	0.708	0.511–0.905	0.038*	100.0	61.1	53.3	30.1–75.2	100.0	74.1–100.0	–	–	–
FF A4 Phase 3	≥17.237	0.634	0.374–0.894	0.313	42.9	93.8	75.0	30.1–95.4	78.9	56.7–91.5	11.3	0.91–139.49	0.067^§^
Serum aromatase Phase 1	≥0.15	0.529	0.303–0.755	0.233	60.0	63.2	46.2	21.5–72.7	75.0	50.5–89.8	2.6	0.53–12.38	0.270
Serum aromatase Phase 2	≥0.059	0.510	0.273–0.747	0.292	88.9	29.4	40.0	22.7–60.4	83.3	43.6–96.9	3.3	0.33–34.12	0.380
Serum aromatase Phase 3	≥0.252	0.694	0.383–1.006	0.164	75.0	66.7	50.0	21.5–78.5	85.7	48.7–97.4	6.0	0.42–85.25	0.266
FF aromatase Phase 3	≤0.378	0.727	0.482–0.971	0.007^†^	75.0	81.3	66.7	35.4–87.9	86.7	60.6–96.4	13.0	1.70–99.38	0.021*
Serum E2 Phase 1	≤0.034	0.688	0.472–0.904	0.088^§^	60.0	88.2	75.0	35.6–95.5	78.9	54.4–93.9	11.3	1.61–78.57	0.025*
Serum E2 Phase 2	≤1.120	0.512	0.279–0.746	0.917	66.7	50.0	40.0	16.3–67.7	75.0	42.8–94.5	2.0	0.38–10.58	0.683
Serum E2 Phase 3	≥1.068	0.713	0.491–0.935	0.061^§^	66.7	77.8	60.0	26.2–87.8	82.4	56.6–96.2	7.0	1.19–41.36	0.039*
FF E2 Phase 3	≥162.97	0.676	0.413–0.940	0.190	71.4	66.7	50.0	21.1–78.9	83.3	51.6–97.9	5.0	0.70–35.50	0.172

AUC, area under the receiver operating characteristic (ROC) curve; PPV, positive predictive value; NPV, negative predictive value; A4, androstenedione; E2, estradiol; FF, follicular fluid; FET, Fisher’s exact test; SN, sensitivity; SP, specificity; Phase 1, beginning of recombinant FSH (rFSH) stimulation; Phase 2, approximately 8 days after rFSH stimulation; Phase 3, on the day of ovum pick-up (OPU).

* *P* < 0.05.

^†^ *P* < 0.01.

^§^ *P* indicates a trend toward significance.

FF aromatase in Phase 3, at a cutoff of ≤ 0.378 ng/mL, exhibited strong predictive performance (AUC = 0.727, *P* = 0.007) and was significantly associated with the outcome, with an odds ratio of 13.0 (Fisher’s exact test *P* = 0.021) (Table 4).

For E2, serum E2 in Phase 1 at a cutoff ≤ 0.034 ng/mL showed borderline discriminatory performance (AUC = 0.688, *P* = 0.088), with a significant association indicated by an odds ratio of 11.3 (Fisher’s exact test *P* = 0.025) (Table 4). In addition, serum E2 in Phase 3 at a cutoff ≥ 1.068 ng/mL also demonstrated a borderline discriminatory performance (AUC = 0.713, *P* = 0.061), with an odds ratio of 7.0 (Fisher’s exact test *P* = 0.039) (Table 4).

#### Ratios of E2, kisspeptin, FSH, and IGF1 treatment concentrations in KGN cells compared to serum or FF in women undergoing IVF/ICSI treatment

Ratios of E2, kisspeptin, FSH, and IGF1 treatment concentrations in KGN cells compared to serum or FF in women undergoing IVF/ICSI treatment are presented in Supplement Table 1 (see section on Supplementary materials given at the end of the article).

The concentrations of E2 used in KGN cells were markedly higher than serum E2 concentrations across all phases of IVF/ICSI treatment, with ratios ranging from approximately 320- to 60,000-fold, depending on dose and phase. In contrast, the differences relative to FF concentrations were substantially smaller, ranging from approximately 2.5- to 13.7-fold.

Kisspeptin concentrations were consistently higher than both serum and FF levels, with ratios of approximately 14- to 27-fold. In contrast, FSH and IGF1 concentrations were substantially lower than physiological levels, with ratios remaining below 0.02 across all phases.

Overall, these findings indicate that the E2 and kisspeptin concentrations used in KGN cell experiments represented supraphysiological exposure relative to their corresponding serum and FF concentrations, whereas FSH and IGF1 were present at sub-physiological levels relative to serum and FF.

### KGN cell experiments

#### Steroidogenic gene expression analysis in KGN cells

**Figure 4:**
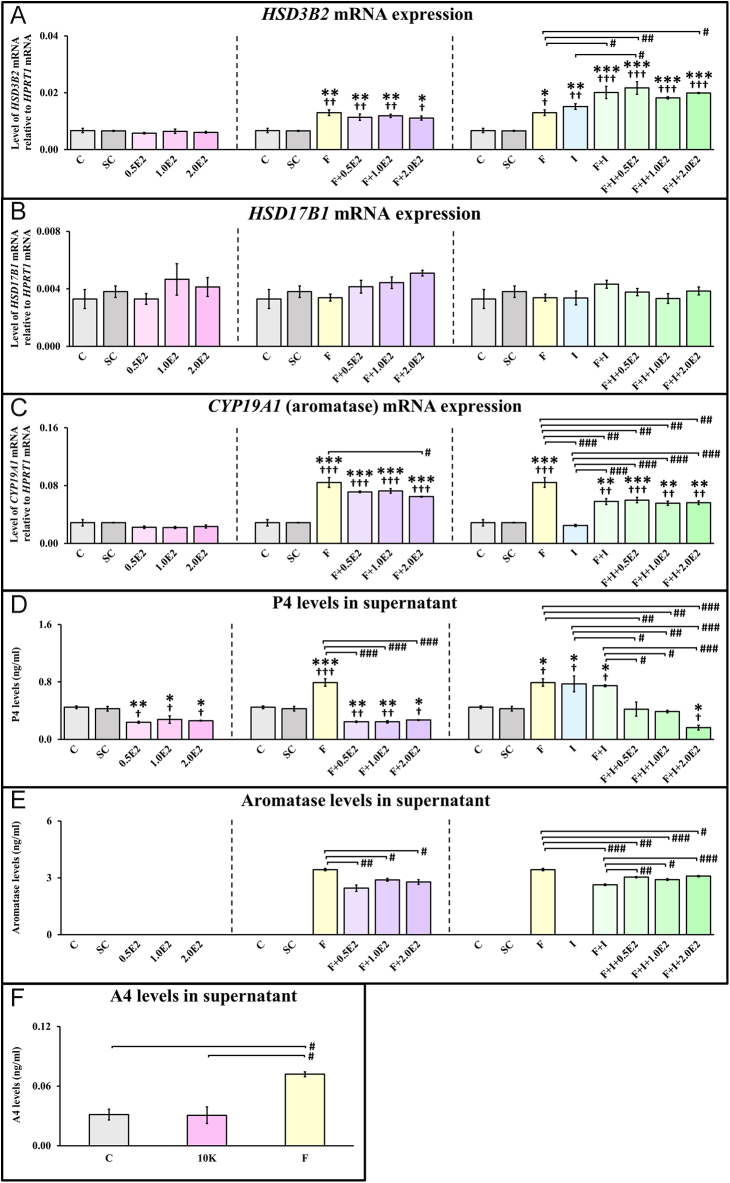
KGN cell experiments. (A) hydroxy-5-steroid dehydrogenase*,* 3 beta- and steroid delta-isomerase 2* (HSD3B2)*, (B) hydroxysteroid 17-beta dehydrogenase 1* (HSD17B1)*, and (C) cytochrome P450 family 19 subfamily A member 1 (*CYP19A1*; aromatase) mRNA expression levels in KGN cells, as well as (D) progesterone (P4), (E) aromatase, and (F) androstenedione (A4) levels in the culture supernatant. mRNA expression levels were normalized to hypoxanthine phosphoribosyltransferase 1* (HPRT1)*. Data are presented as mean ± SEM. Statistical significance is indicated as *P < 0.05, **P < 0.01, ***P < 0.001 compared with control (C); ^†^*P* < 0.05, ^††^*P* < 0.01, ^†††^*P* < 0.001 compared with solvent control (SC); and ^#^*P* < 0.05, ^##^*P* < 0.01, ^###^*P* < 0.001 between groups. 0.5E2, 1.0E2, 2.0E2: 0.5, 1, 2 μg/mL estradiol, respectively; F, 0.01 nM FSH; I, 0.01 nM insulin-like growth factor 1 (IGF1); 10K, 10 μM kisspeptin.

The effects of E2, FSH, and/or IGF1 treatments on *HSD3B2*, *HSD17B1*, and *CYP19A1* gene expression in KGN cells are presented in Fig. 4A, B, C. FSH significantly increased *HSD3B2* mRNA expression compared with control and solvent control groups (*P* < 0.01) (Fig. 4A). IGF1 alone also increased *HSD3B2* expression compared with control (*P* < 0.01), and combined FSH + IGF1 treatment further enhanced expression compared with FSH alone (*P* < 0.05; Fig. 4A). The addition of E2 to FSH or FSH + IGF1 did not alter *HSD3B2* expression (Fig. 4A).

FSH significantly increased *CYP19A1* mRNA expression compared with controls (*P* < 0.001; Fig. 4C). High-dose E2 combined with FSH reduced *CYP19A1* expression compared with FSH alone (*P* < 0.05; Fig. 4C). FSH + IGF1 resulted in lower *CYP19A1* expression compared with FSH (*P* < 0.01), and adding E2 to FSH + IGF1 produced no further change (Fig. 4C).

#### Steroid hormone and aromatase analysis in KGN cell culture supernatants

The effects of E2, FSH, and/or IGF1 treatments on P4, aromatase, and A4 levels in KGN cell culture supernatants are presented in Fig. 4D, E, F. E2 at any dose reduced supernatant P4 levels compared with controls (*P* < 0.05; Fig. 4D). FSH alone increased P4 compared with controls (*P* < 0.001), whereas adding E2 at any dose to FSH reduced P4 compared with FSH (*P* < 0.001; Fig. 4D). IGF1 alone increased P4 compared with controls (*P* < 0.05; Fig. 4D). FSH + IGF1 did not increase P4 compared with FSH alone or IGF1 alone (Fig. 4D). Adding E2 at any dose to FSH + IGF1 reduced P4 compared with FSH + IGF1 (*P* < 0.05; Fig. 4D).

Aromatase was detectable only in response to FSH (Fig. 4E). Adding IGF1 or E2 at any dose to FSH reduced aromatase levels compared with FSH alone (*P* < 0.01; Fig. 4E). In contrast, adding E2 at any dose to FSH + IGF1 increased aromatase compared with FSH + IGF1 (*P* < 0.05), but levels remained lower than with FSH alone (Fig. 4E).

Kisspeptin and FSH both increased A4 concentrations in the supernatant compared with control (*P* < 0.05; Fig. 4F), indicating that KGN cells can synthesize and secrete A4 in the absence of theca cells.

## Discussion

This study provides an integrated assessment of phase-specific steroid dynamics during IVF/ICSI and complementary mechanistic evidence from KGN cells. To our knowledge, this is the first study to combine longitudinal clinical measurements with cellular experiments and to link hormone trajectories with potential regulatory mechanisms.

A principal clinical finding of this study is that successful IVF/ICSI cycles are characterized by a coordinated late-follicular rise of both A4 and E2 toward the ovulatory phase (Phase 3). Women who achieved clinical pregnancy exhibited significantly greater Δ and fold changes in serum A4 and E2 from Phase 2 to Phase 3, along with higher Phase 3 steroid-to-basal gonadotropin ratios for both LH and FSH. In addition, serum A4 at Phase 2 positively correlated with serum E2 at Phases 2 and 3 and with key reproductive outcomes, including the number of retrieved and mature oocytes. Serum A4 at Phase 3 was also positively correlated with serum E2 at Phases 2 and 3 and FF E2 at Phase 3. These findings indicate that effective late-follicular amplification of steroidogenesis, rather than absolute hormone concentrations alone, is a hallmark of successful IVF/ICSI cycles.

These observations are consistent with previous clinical studies. Higher serum A4 levels during the late stimulation phase have been reported in pregnant women, particularly among women with PCOS (Swellam *et al.* 2013), and A4 levels on the day of hCG administration have been positively correlated with follicle number, peak E2 levels, oocyte yield, and embryo number (Frattarelli & Gerber 2006). Together, these data support the concept that sufficient androgen substrate availability during the peri-ovulatory period is essential for sustaining optimal estrogen production and follicular maturation.

In contrast, the unsuccessful pregnancy group exhibited a blunted or dysregulated steroid trajectory. Our data demonstrate that this Phase 2–Phase 3 surge is attenuated in unsuccessful cycles, resulting in reduced Δ and fold change values for both A4 and E2. Notably, despite lower estrogen output, FF aromatase levels were not reduced and were, in some cases, relatively higher. The dissociation between FF aromatase levels and E2 suggests that reduced late-phase E2 may relate to substrate availability and/or functional coupling rather than aromatase quantity.

In addition to impaired late-phase steroid rise, our study demonstrates that elevated androgen and estrogen signals during the basal phase (Phase 1) are negatively associated with IVF/ICSI outcomes. Higher basal A4 and E2 levels correlated with fewer retrieved oocytes, fewer mature oocytes, and reduced embryo numbers. Basal serum E2 at Phase 1 also showed negative correlations with favorable reproductive parameters, including serum AMH at Phase 1 and serum LH at Phase 3. ROC analysis further suggested that lower Phase 1 serum E2 levels may be predictive of clinical pregnancy, supporting the concept that restrained early steroid exposure is advantageous.

These findings are supported by prior clinical and experimental evidence. In women with PCOS, lower early-follicular A4 levels have been reported in those who achieved pregnancy (Kahyaoglu *et al.* 2023). Animal studies further demonstrate that excessive androgen exposure during the early follicular phase disrupts granulosa cell function, induces oocyte abnormalities, increases apoptosis, promotes follicular cyst formation, and leads to premature luteinization (Okutsu *et al.* 2010, Tarumi *et al.* 2012).

KGN cells were selected because they retain several functional characteristics of human granulosa cells, including steroidogenic activity and responsiveness to gonadotropins, thereby providing a relatively stable and reproducible *in vitro* model for mechanistic studies of ovarian steroidogenesis (Nishi *et al.* 2001). However, KGN cells originate from an adult granulosa cell tumor and harbor the *FOXL2* C134W missense mutation (Jamieson *et al.* 2010), which may influence granulosa cell differentiation and steroidogenic responses. Therefore, the findings from KGN cells in the present study should be interpreted as mechanistic and hypothesis-generating rather than direct representations of normal human granulosa cell physiology *in vivo*.

Importantly, although primary human granulosa cells obtained during IVF/ICSI procedures may provide additional translational relevance, these cells are also associated with substantial experimental limitations (Havelock *et al.* 2004, Hensen *et al.* 2020, Zgórecka *et al.* 2022). Granulosa cells collected at oocyte retrieval have already been exposed *in vivo* to controlled ovarian hyperstimulation protocols, including rFSH stimulation, GnRH antagonist treatment, and hCG ovulatory triggering before follicular aspiration (Havelock *et al.* 2004). Such interventions may alter granulosa cell differentiation status, luteinization, steroidogenic activity, and transcriptional responses (Havelock *et al.* 2004, Hensen *et al.* 2020, Zgórecka *et al.* 2022). In addition, primary granulosa cell cultures are typically obtained in limited quantities, may not survive through extended culture periods (Hensen *et al.* 2020, Zgórecka *et al.* 2022), and often require exogenous hormonal supplementation, growth factors, serum-free optimization protocols, and variable *ex vivo* culture conditions to maintain viability and proliferation *in vitro* (Hensen *et al.* 2020, Zgórecka *et al.* 2022), all of which may further influence cellular physiology and contribute to inter-sample heterogeneity. Previous studies have also demonstrated that steroidogenic characteristics and cellular behavior of granulosa cells may vary according to culture systems, hormonal conditions, and follicular developmental stage (Havelock *et al.* 2004, Hensen *et al.* 2020, Zgórecka *et al.* 2022). Therefore, although primary granulosa cells are clinically relevant, interpretation of their *in vitro* responses also requires caution.

Mechanistic insights from KGN cell experiments demonstrated that IGF1 increased *HSD3B2* mRNA expression and P4 levels in the culture supernatant, both of which may represent steroidogenic processes preceding androgen formation (early-step steroidogenesis). IGF1 was included in the KGN cell experiments based on our previous finding that kisspeptin enhanced FSH-induced *CYP19A1* expression in KGN cells only in the presence of IGF1, whereas FSH alone did not significantly alter *CYP19A1* expression (Qin *et al.* 2021). Furthermore, IGFI has been shown to augment FSH-induced expression of *Star*, *Hsd3b2*, and *Cyp11a1* in primary rat granulosa cells, increase *Cyp19a1/CYP19A1* expression in primary mouse and human granulosa cells (Zhou *et al.* 2013), and potentiate FSH-stimulated P4 and E2 production in primary human granulosa cells (Erickson *et al.* 1989, 1991).

In contrast, FSH stimulated *HSD3B2* mRNA expression and P4 and A4 levels in the culture supernatant, together with *CYP19A1* mRNA expression and detectable aromatase levels, the latter of which may represent steroidogenic processes occurring subsequent to androgen formation (late-step steroidogenesis).

Aromatase (CYP19A1) is generally recognized as an intracellular, membrane-bound enzyme localized predominantly in the endoplasmic reticulum, where it catalyzes estrogen biosynthesis from androgen precursors (Adhikari *et al.* 2017). Therefore, aromatase is not classically considered a secreted protein. However, measurable aromatase levels have been reported in extracellular biological samples, including serum (Manwani *et al.* 2021) and cell culture supernatants (Qin *et al.* 2021). In the present study, aromatase levels in KGN cell culture supernatants were detectable only following FSH treatment, whereas levels in the absence of FSH remained below the assay detection range. Similarly, serum aromatase levels were detectable only in a subset of participants undergoing IVF/ICSI treatment. These findings suggest that extracellular detectable aromatase protein may be present under certain physiological or experimental conditions. Potential mechanisms may include passive extracellular release during cellular stress, cell injury, apoptosis, or membrane damage (Murao *et al.* 2021), as well as extracellular vesicle/exosome-associated transport and membrane shedding processes that can transfer membrane-associated proteins into the extracellular environment (Raposo & Stoorvogel 2013). Therefore, detectable aromatase levels in the present study may reflect extracellular presence or accumulation of aromatase protein rather than classical secretion.

In this study, IGF1 was selected instead of insulin like growth factor 2 (IGF2) because IGF1 levels were evaluated in serum across all three IVF/ICSI phases and in FF during Phase 3 on the day of ovum pick-up (OPU), enabling comparison between physiological concentrations observed during IVF/ICSI treatment and the IGF1 treatment concentrations used in KGN cell experiments. In contrast, FF samples could only be collected during Phase 3 on the day of OPU, because FF aspiration during earlier phases would be invasive and could potentially interfere with IVF/ICSI treatment outcomes. In addition, IGF1 has been more extensively characterized in relation to ovarian folliculogenesis, granulosa cell function, steroidogenesis, and IVF/ICSI outcomes (Silva *et al.* 2009, Faraj *et al.* 2017, Scheffler *et al.* 2021). In contrast, IGF2 is considered a major and highly abundant IGF within the FF microenvironment and has also been implicated in follicular growth, granulosa cell proliferation, steroidogenesis, oocyte maturation, and developmental competence (Afradiasbagharani *et al.* 2022). Nevertheless, because IGF2 was not measured in the present study, the current findings may not fully represent the complexity of the IGF system within the follicular microenvironment.

E2 concentrations of 0.5, 1.0, and 2.0 μg/mL were chosen based on previous evidence demonstrating that E2 at concentrations of 0.25–2.0 μg/mL reduced basal P4 production and suppressed hCG-stimulated P4 secretion in human granulosa-luteal cells (Jalkanen 1990), suggesting that this concentration range may influence ovarian steroidogenesis. At the time of experimental design, however, it remained unclear whether these treatment concentrations corresponded to physiological or supraphysiological E2 exposure relative to levels present in human serum and FF. Although a previous study reported that 1,000 nM E2 (approximately 0.27 μg/mL) represented a supraphysiological concentration in KGN cells (Haltia *et al.* 2020), the basis for this classification was not specified. Given that the E2 concentrations used in the present study exceeded this previously reported level, it was anticipated that they might similarly represent excessive E2 exposure and serve as a model of E2 supplementation. Subsequent comparison with serum and FF E2 concentrations measured in our IVF/ICSI cohort demonstrated that the E2 treatment concentrations were approximately 2–4, 5–7, and 10–14 times higher than physiological FF E2 concentrations, respectively, suggesting that these concentrations represented supraphysiological exposure.

E2 at concentrations of 0.5, 1.0, and 2.0 μg/mL suppressed downstream steroidogenic output, particularly aromatase and P4 levels in the culture supernatant, whereas the highest concentration (2.0 μg/mL) additionally reduced *CYP19A1* mRNA expression. These effects were further enhanced when E2 was combined with FSH and/or IGF1. These cellular findings align with the concept that elevated early-follicular-phase E2 concentrations constrain later steroidogenic capacity by suppressing key steroidogenic pathways, underscoring that E2 is not merely an end product of steroidogenesis but also a dose-dependent regulator of steroidogenic flux.

Remarkably, although androgens are classically considered to be synthesized predominantly in theca cells, A4 was detected in the supernatant of KGN cells even in the absence of theca cells and exogenous androgen substrates. This observation is consistent with a potential contribution of granulosa cells to local androgen availability under certain conditions. Baseline androgen concentrations in the culture media and serum supplements were not measured; however, KGN cells were initially maintained in complete medium containing 10% FBS before treatment, whereas all subsequent treatments were performed in serum-free DMEM/F-12, which does not contain androgenic steroids according to the manufacturer’s formulation. Therefore, any contribution from trace steroid hormones present in FBS would most likely be limited to basal conditions. In contrast, the significant increase in A4 concentrations following FSH stimulation compared with control conditions indicates an active steroidogenic response in KGN cells. Collectively, these findings suggest that the observed A4 production is unlikely to be attributable solely to residual steroids from culture conditions and is consistent with active androgen production by KGN cells.

Several limitations should be acknowledged. First, the small sample size reduces statistical power, especially for subgroup and ROC analyses. Although phase-specific patterns and biologically coherent associations were observed, the findings require cautious interpretation and validation in larger independent cohorts. Second, successful pregnancy outcomes in IVF/ICSI are multifactorial and may also be influenced by paternal and male factor parameters that were not comprehensively evaluated in the present study. Therefore, the observed associations between steroid dynamics and clinical pregnancy should not be interpreted as exclusively attributable to female endocrine factors alone. Third, serum aromatase was detectable in only a subset of samples, limiting the reliability of systemic assessment. Furthermore, aromatase measurements were based on protein immunoassays and therefore may not fully represent functional enzymatic activity at the cellular level. Future studies simultaneously assessing aromatase protein expression and enzymatic activity in cells are needed to confirm and extend these findings. Fourth, although IGF1 was evaluated because serum measurements could be obtained longitudinally throughout all three IVF/ICSI phases and compared with FF levels obtained during OPU, IGF2, which is considered a major IGF in FF, was not measured in the present study. Therefore, the current findings do not fully represent the IGF system within the follicular microenvironment, and future studies simultaneously assessing IGF1 and IGF2 are warranted. Fifth, baseline androgen concentrations in the culture media and serum supplements were not measured. Although androgenic steroids were not listed as components of the DMEM/F-12 medium according to the manufacturer’s formulation and all treatments were performed under serum-free conditions, KGN cells were initially maintained in complete medium containing 10% FBS before treatment. Therefore, a contribution from trace steroid hormones derived from serum cannot be completely excluded. Finally, the KGN granulosa-like tumor cell line was used instead of primary human granulosa cells. Although KGN cells are widely used as a model for granulosa cell steroidogenesis, they may not fully reproduce the phenotype and steroidogenic responses of primary granulosa cells obtained from IVF patients. In addition, KGN cells originate from an adult granulosa cell tumor and harbor the *FOXL2* C134W mutation, which may influence granulosa cell differentiation and steroidogenic responses. Therefore, the cellular findings should be interpreted cautiously and validated in future studies using primary human granulosa cells.

In conclusion, successful IVF/ICSI cycles were characterized by a favorable phase-specific steroid trajectory, with relatively low basal steroid exposure in Phase 1 followed by a robust late-follicular to ovulatory increase from Phase 2 to Phase 3 in both A4 and E2. This coordinated rise was reflected by greater phase-to-phase differences and fold changes, together with higher Phase 3 steroid-to-basal gonadotropin ratios in women who achieved clinical pregnancy. In contrast, elevated early-follicular Phase 1 E2 levels represented an unfavorable endocrine pattern and were negatively associated with IVF/ICSI outcomes in correlation and ROC analyses. Mechanistically, KGN cell experiments demonstrated that IGF1 enhanced early-step steroidogenesis and FSH activated both early- and late-step pathways, whereas supraphysiological E2 suppressed downstream steroidogenic output. These cellular findings complement the clinical associations between early E2 patterns and subsequent steroidogenic responses. Remarkably, the detection of A4 in KGN cell supernatants in the absence of theca cells and exogenous androgen substrates supports the possibility of granulosa cell contribution to local androgen availability under the absence of theca cells or exogenous androgen substrates *in vitro* conditions characterized by the absence of theca cells and exogenous androgen substrates, particularly following FSH stimulation.

## Supplementary materials



## Declaration of interest

The authors declare that there is no conflict of interest that could be perceived as prejudicing the impartiality of the research reported.

## Funding

This study was supported by the Siriraj Research Fund, Grant number (IO) R016433005, Faculty of Medicine Siriraj Hospital, Mahidol University; the Siriraj Research Development Fund, Grant number (IO) R016734002, Faculty of Medicine Siriraj Hospital, Mahidol University; and Mahidol University, Grant number (IO) R016421007.

## Author contribution statement

RS and CS conceived the study, performed experiments, analyzed data, acquired funding, and wrote the manuscript. IK and SC performed experiments, analyzed data, acquired funding, and wrote the manuscript. PM performed experiments, contributed resources, and wrote the manuscript. SP conceived the study, performed experiments, analyzed data, contributed resources, and wrote the manuscript.
