# Supplementary material for: Late-follicular androgen and estrogen rise associated with clinical pregnancy in IVF/ICSI with granulosa cell studies
Source: Reprod Fertil. 2026 Jul 30;7(3):RAF260062. doi: 10.1530/RAF-26-0062 (PMC13452035; doi:10.1530/RAF-26-0062)
Supplement: Supplementary file 1 [file RAF-26-0062_supplementary_table_1.pdf]

Supplement Table 1

Ratios of estradiol (E2), kisspeptin, follicle-stimulating hormone (FSH), and insulin-like growth factor 1 (IGF1) treatment concentrations in human granulosa-like tumor (KGN) cells compared to serum or follicular fluid (FF) in women undergoing IVF/ICSI treatment.

Subs-tances	Concen-trations	Change to ng/mL	Serum Phase 1		Serum Phase 2		Serum Phase 3		FF Phase 3	
			Success	Unsuccess	Success	Unsuccess	Success	Unsuccess	Success	Unsuccess
0.5E2	0.5 µg/mL	500	15,146.93	11,266.34	320.92	391.57	373.74	557.03	2.51	3.42
1.0E2	1.0 µg/mL	1,000	30,293.85	22,532.67	641.84	783.14	747.48	1,114.07	5.01	6.83
2.0E2	2.0 µg/mL	2,000	60,587.70	45,065.34	1,283.69	1,566.28	1,494.97	2,228.14	10.02	13.66
10K	10 µM	13.024	24.67	23.30	21.86	24.91	23.10	26.90	14.57	23.96
F	0.01 nM	0.00036	0.000056	0.000041	0.000027	0.000020	0.000036	0.000037	-	-
I	0.01 nM	7.6×10^{-7}	0.0067	0.0064	0.0099	0.0168	0.0083	0.0046	0.0042	0.0027

Phase 1, before recombinant follicle-stimulating hormone (rFSH) stimulation; Phase 2, approximately 8 days after rFSH stimulation; Phase 3, on the day of ovum pick-up (OPU); 0.5E2, 0.5 µg/mL estradiol; 1.0E2, 1 µg/mL estradiol; 2.0E2, 2 µg/mL estradiol; F, 0.01 nM FSH; I, 0.01 nM IGF1; FSH was not measured in follicular fluid (FF); therefore, ratios of FSH concentrations in KGN cells compared with FF in women undergoing IVF/ICSI treatment are not available.
